# Exploiting Self-organization in Bioengineered Systems: A Computational Approach

**DOI:** 10.3389/fbioe.2017.00027

**Published:** 2017-04-28

**Authors:** Delin Davis, Anna Doloman, Gregory J. Podgorski, Elizabeth Vargis, Nicholas S. Flann

**Affiliations:** ^1^Computer Science Department, Utah State University, Logan, UT, USA; ^2^Department of Biological Engineering, Utah State University, Logan, UT, USA; ^3^Department of Biology, Utah State University, Logan, UT, USA

**Keywords:** agent-based modeling, multicellular modeling, self-organization, vasculogenesis, biomanufacturing

## Abstract

The productivity of bioengineered cell factories is limited by inefficiencies in nutrient delivery and waste and product removal. Current solution approaches explore changes in the physical configurations of the bioreactors. This work investigates the possibilities of exploiting self-organizing vascular networks to support producer cells within the factory. A computational model simulates *de novo* vascular development of endothelial-like cells and the resultant network functioning to deliver nutrients and extract product and waste from the cell culture. Microbial factories with vascular networks are evaluated for their scalability, robustness, and productivity compared to the cell factories without a vascular network. Initial studies demonstrate that at least an order of magnitude increase in production is possible, the system can be scaled up, and the self-organization of an efficient vascular network is robust. The work suggests that bioengineered multicellularity may offer efficiency improvements difficult to achieve with physical engineering approaches.

## 1 Introduction

Recent developments in genetics, bioengineering, synthetic biology, and nanotechnology have enabled industrial scale biomanufacturing units for the production of many valuable products (Sharma et al., 2001). Biopharmaceutical and recombinant enzyme proteins production is of primary importance within this field (van Dijl and Hecker, 2013). Insulin (Walsh, 2005); vanillin (Hansen et al., 2009; Brochado et al., 2010); antibodies such as penicillin (Kiel et al., 2005); and industrially valuable enzymes such as lipase (Pandey et al., 1999), cellulase (Kuhad et al., 2011), and amylase (Pandey et al., 2000) are already being produced in large scale with microbial factories. Apart from enzyme production, microbes are also being engineered to produce industrially important nanoparticles that are used in electronics and drug delivery (Dhanjal and Cameotra, 2010; Villaverde, 2010), environmentally beneficial bioplastics (Höfer et al., 2011), insect silk (Scheibel, 2004), opiates (Thodey et al., 2014), biofuels such as isobutanol (Desai et al., 2015), various chemicals (Vázquez et al., 2010), and proteins (Ferrer-Miralles et al., 2009). These industrial bioreactors are relatively inexpensive and efficient.

The quality and composition of the medium in which these cell factories are cultivated significantly influences the productivity of the system (Hahn-Hägerdal et al., 2005). The optimal medium composition varies between species and strains. For example, *Escherichia coli* is often cultured in a fed-batch operation for recombinant protein production (Losen et al., 2004; Farrell et al., 2015; Sohoni et al., 2015). As the producer cells grow, they not only create the product but also produce waste. The accumulation of waste can have adverse effects on the culture, degrade product quality, and make it difficult to isolate the product (Hahn-Hägerdal et al., 2005). For example, the acetate that inevitably forms during fermentation of glucose by *E. coli* inhibits further glucose fermentation. Overcoming the issue of waste accumulation even at low nutrient concentrations is a significant concern for recombinant protein production (Eiteman and Altman, 2006; Sanchez-Garcia et al., 2016). Diffusion of nutrients and oxygen is rate limiting for many aerobic fermentations (Sandén et al., 2003). Moreover, the accumulation of the product can act in a negative feedback loop and inhibit the production of further product (Aiba et al., 1968; Levenspiel, 1980; Han and Levenspiel, 1988).

Frequent efforts to increase the stability and productivity of cellular factory designs focus on controlling the cell culture during production. The microenvironment of the factory cells must be kept free of waste and product and continually replenished with nutrients. Closed fed-batch systems achieve this by dispersing the waste and product throughout the bioreactor by agitation, while in chemostats, flow-through processes ensure that the cell microenvironments are regularly replaced with new media. This work explores a possible alternative solution to the problem of controlling and optimizing the culture for product production.

Multicellular organisms depend on vascular systems for nutrient delivery and waste removal (Monahan-Earley et al., 2013). These vascular networks are formed either through vasculogenesis, a biological process in which scattered vessel precursor cells self-organize to form new networks, or through angiogenesis, in which new vessels sprout from the existing vessels.

Both vasculogenesis and angiogenesis are driven primarily by chemotaxis, a mechanism in which cells move in response to a chemical gradient, along with cell–cell adhesion (Merks et al., 2008). While many questions remain, progress in understanding and exploiting both vasculogenesis and angiogenesis is being made from a bioengineering perspective (Kaully et al., 2009; Lovett et al., 2009). Takebe and colleagues (Takebe et al., 2014) successfully implanted tissue-engineered vascular grafts in baboons and dogs. Melero-Martin et al. (2008) showed that robust development of functional vascular networks is possible *in vivo*. With additional research in this area, bioengineered cells could be used to form functional vascular networks to create a useful delivery mechanism in cell factories. The producer cells bounded by the vascular networks would serve as biocatalysts, converting supplied nutrients into a product, which is in turn transported by vascular cells away from the producer cells. Previous efforts to grow cells as biocatalysts have proved viable if cells are also encapsulated in a carrier material that acts as a semipermeable membrane (Pscheidt and Glieder, 2008; Hasunuma and Kondo, 2012).

This article presents a proof-of-concept cellular factory design in which the producer cell cultivation environment is supported by a self-organizing vascular network replicating the nutrient delivery and waste removal process in multicellular organisms. A simulation study evaluates the potential of the design for enhanced production from stable cultures. In this design, the vascular networks self-organize from randomly distributed bioengineered cells with properties similar to endothelial cells. These newly formed vessels then support the product-producing cells by delivering nutrients, extracting product, and removing waste.

## 2 Multicellular Experiment and Model

**Figure 1:**
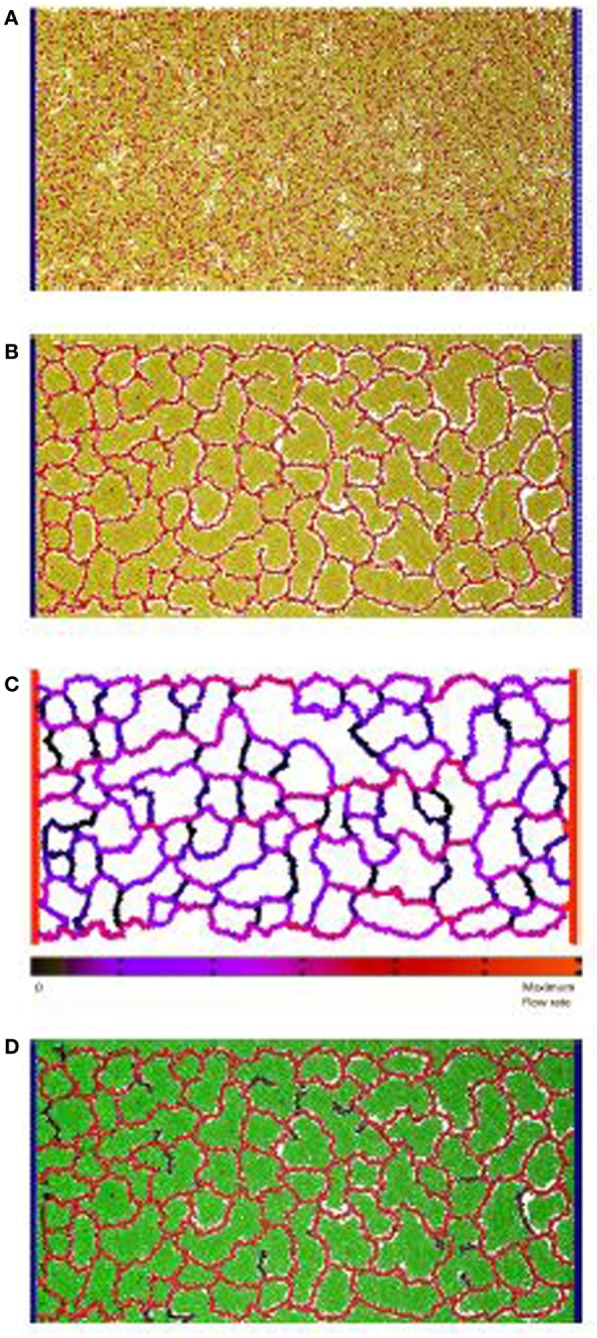
**Vascular factory: the three phases in modeling a vascular factory design**. Beginning with morphogenesis, where the vessel network self-organizes, followed by network analysis where the flow through the vessels is estimated, and then production, where the producer cells run and the nutrient inflow and the product outflow are calculated. **(A)** *Initial state*: random arrangement of vascular cells (red spheres) and inactive producer cells (yellow spheres). The fixed circulatory cells are shown in blue on both sides. **(B)** *Phase I*: self-organize the vessel network. Each vascular cell secretes and responds to a chemoatractant. Strong mutual adhesion builds vessels that connect into a near-regular network, partitioning the producer cells in each lacunae. **(C)** *Phase II*: determine network flow. A network of pipes is extracted and a pressure differential applied (source and sink). Individual vessel flow is determined by applying Poiseuille’s Law (red is high, black is low). **(D)** *Phase III*: run the factory. Nutrient flows into the network and is dispersed, to activate the producer cells (green spheres). The network removes the product, which flows to the sink.

An overview of the simulation process that builds and executes the novel design for a microbial cell factory is given in Figure 1. Here, a self-organizing vascular network is formed that provides nutrient delivery and removal of product and waste from the factory cells that produce the desired product. The cell factory is created in three distinct phases: self-organization of the vascular network, initiation of material flow through the vascular network, and finally, the production of product and removal of waste in the factory.

At this proof-of-concept stage, a two-dimensional model was constructed and evaluated. Figure 1A illustrates the initial state of the simulated factory. Vessel cells (blue) that simulate the external circulatory system are arranged in two columns on both sides of the production area. The left column represents the source, and the right column represents the sink, in parallel to the arterial–venous network architecture in vertebrates (Reiber and McGaw, 2009). Circulatory cells are capable of secreting both long-range and short-range chemoattractants to enable the self-organized, newly formed vessels to connect in the circulatory system. Inactive producer cells (yellow) are randomly distributed across the area in between the two circulatory cell columns and are mixed with bioengineered vascular cells (red), similar to the endothelial cells in vertebrates (Merks et al., 2008).

In the first phase, the randomly distributed vascular cells self-organize to form a vascular network that is connected to both columns of circulatory cells. This process partitions the producer cells into clusters contained within each network lacuna. An example of a self-organized system at the end of Phase I is illustrated in Figure 1B. In the second phase, flow through the vascular system is estimated. Figure 1C shows the color-coded magnitude of the calculated flow. Finally, the factory is executed. Here, the functioning vascular network simulation is integrated with producer cells that consume nutrients and secrete product to simulate the steady-state operation of the cell factory. The running factory is illustrated in Figure 1D. Note that in this case, the network provides almost complete coverage of the producing area, and all cells are active (green).

## 3 Evaluation and Results

Thousands of simulations were performed to gain insights into the feasibility and potential benefits of this vascular factory design. Three specific questions were addressed. First, how is the productivity of the factory affected by the proportion of vascular cells initially introduced into the factory? Second, how robust is the self-organizing process? Robustness was investigated by generating multiple vascular networks under different random starting conditions. Finally, how do the physical dimensions of the factory area influence productivity? Here, the influence of separating the two circulatory columns over a range of distances was explored.

Due to computational resources limitations, the size of the factory is restricted to a fixed height of 516 μm and widths that varied from 516 μm to 2.054 mm. Each simulation has 780 circulatory cells arranged as two vessels (columns) on either side of the grid to simulate the external delivery and extraction systems. The total numbers of vascular and producer cells were set to 12,000, 24,000, or 48,000. At the beginning of each execution, vascular and producer cells are randomly distributed in the area between the circulatory cell columns (see Figure 1A). In Phase III, the throughput of the system is calculated by summing the total product removed by the vascular network over two simulated hours once the factory has reached a steady state.

**Figure 2:**
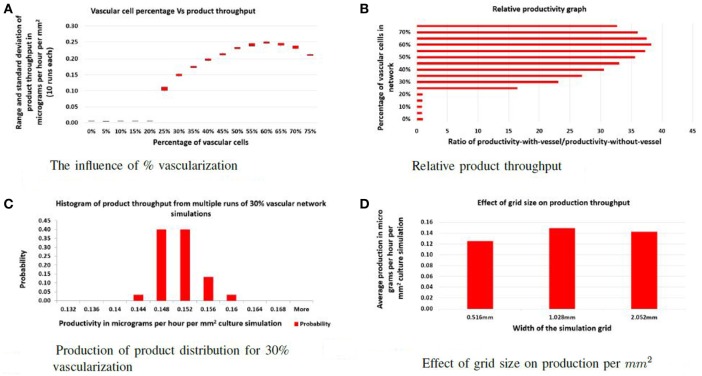
**Evaluation: (A) the effect of varying the percentage of vascular cells in the initial random configuration on the total product throughput per hour; (B) the effect of varying the percentage of vessel cells in the initial random configuration on relative productivity**. Productivity is measured by dividing throughput of the specific experiment with the throughput of the factory with no vascular system; **(C)** illustration of the robustness of the self-organizing process as a histogram of product throughput from networks generated with different random seeds; **(D)** the effect of increasing the width of the culture. Here, the actual productivity is given in micrograms per hour per cubic micrometer of culture.

**Figure 3:**
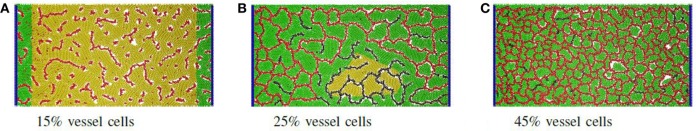
**Proportion of vascular cells: example of self-organized networks from different proportions of vessel and producer cells**. **(A)** At 15%, only isolated and dysfunctional vessels are formed. **(B)** At 25%, regions with disconnected vessels become common, leading to areas of inactive producer cells (yellow). In this case, darker vascular cells signify very low or no nutrient flow. **(C)** At higher densities such as 45%, many small lacunae are formed and all producer cells are active.

To address the first question, the ratio of vascular cells to producer cells was varied between 0 and 75% while keeping the total number of cells constant. The factory dimensions were fixed at 516 μm by 1.028 mm. For each ratio, 10 simulations were performed, and the average product throughput calculated. In Figure 2A, the productivity is measured in micrograms of product per hour that passed to the sink. Figure 2B shows product throughput measured relative to the throughput when no vasculogenesis occurs. When the proportion of vascular cells is below 20%, the vascular network never completes a path between the two circulatory columns, and no advantage in production is realized. Between 20 and 25% vascular cells, the self-organized networks are not robust: few of these connect between the source and sink vessel elements, and most contain regions of disconnected vessels that leave regions of producer cells without support. At 30%, the vascular network demonstrates few defects, and vessels are well distributed and cover the entire area of producer cells. Typical examples of networks at different vessel/producer cell ratios are illustrated in Figure 3.

Simulations with functional vascular networks produced a 15- to 40-fold increase in the product produced compared to simulations run without vascular systems (see Figure 2B). With functional vascular systems, relative productivity increases and maximizes at 60% vascular-to-producer cell ratio and then reduces as vascular density crowds out the producer cells. These experiments show that vascular networks increase individual cell productivity and thereby the overall efficiency of the factory.

For this approach to be viable, the self-organizing step that builds the vascular system must be insensitive to the initial random arrangement of cells and the stochasticity of cell movement. In each vascular factory, an effective network that can deliver nutrients and collect product must be constructed autonomously. In the second set of experiments, the ratio of vascular and producer cells is fixed at 30%, and multiple simulations were performed, each with a unique random seed. As shown in Figure 2C, although the vascular networks formed in each case were distinct, all functioned similarly and produced little variability in overall production. Hence, the self-organization process is robust.

**Figure 4:**
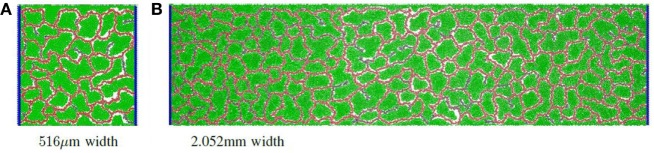
**Simulation examples with different grid widths: (A) an example of a vascular network with grid size 516 μm × 516 μm; (B) an example of a vascular network with grid size 516 μm × 2.052 mm**.

Finally, the separation distance of circulatory columns was explored. Ideally, circulatory columns would be widely spaced, with the self-organized network supporting the bulk of the producer cells needs. In this study, the height was maintained at 516 μm, and the width varied between 516 μm and 2.054 mm with widths changed by powers of two. Figure 2D illustrates the productivity estimates for three different widths. These results show that the productivity per unit area varied little with changes in width. Therefore, productivity improves linearly with separation distance, suggesting that a design with a small number of widely separated pipes (vessels) will provide maximal product production. Examples of a vascular network for the 516 μm and 2.054 mm widths are given in Figure 4A and Figure 4B, respectively.

## 4 Materials and Methods

An agent-based simulator framework, *cDynoMiCs* (Baker et al., 2015) was used in this study. *cDynoMiCs* is an extension of *iDynoMiCs* framework developed by the Kreft group at University of Birmingham (Lardon et al., 2011) for investigations of biofilms. *cDynoMiCs* facilitates modeling eukaryotic cells with the addition of extracellular matrix, tight junctions, and chemotaxis. Each cell is represented as a spherical particle, which has a particular biomass with cell type-specific properties. Particles can secrete or take up chemicals that diffuse through the domain. Particles also exhibit homogeneous and heterogenous adhesion, chemotaxis, and the formation of tight junctions. The simulation process interleaves biomechanical stress relaxation where the particles are moved in response to individual forces and biochemical processes such as secretion, uptake, and diffusion of molecules through a differential equation solver.

### 4.1 Phase I: Self-organize the Vascular Network

Beginning in the randomly seeded state such as that illustrated in Figure 1A, the vessel and circulatory cells become active and start to secrete chemoattractants, described by the Monod-kinetic reaction in equation (1). *N_c_* is the concentration of the nutrient initially supplied for chemoattractant (C) secretion, *M* is the mass of the vascular or circulatory cell, and the diffusion coefficient, *D_c_*, of both chemoattractants are set to 1 × 10^−13^ m^2^ s^−1^ as given in the *in vitro* angiogenesis study of Merks et al. (2008). The chemoattractant secreted by the vascular cells has a fast rate of decay, β*_v_*, which creates a steep local gradient surrounding each cell. The chemoattractant secreted by the circulatory cells β*_c_* has a slower rate of decay creating a longer range gradient.

(1) $$\frac{\partial C}{\partial t}=D_{c}{▿}^{2}C+\mu_{c}\frac{k}{\left(N_{c}+k\right)}M_{v}-\beta C.$$

The vascular cells respond to the gradient of the chemoattractants by tending to move “uphill,” a process described in equation (2) and by Adler (1965). Let *p* be a particle that responds to chemoattractant *C*. A random unit vector $\vec{c}$ is generated and considered as a potential chemotactic force on *p*. The local gradient of chemoattractant across *p* in direction $\vec{c}$ is determined by sampling *C* ahead of *p*, referred to as *C*^+^, and behind *p*, referred to as *C*^−^. The magnitude of force Δ*F* in direction $\vec{c}$ is given by equation (2) (Merks et al., 2008), where λ is the parameter that controls the magnitude of the response to the gradient.

(2) $$F=\lambda \;\left(\frac{C^{+}}{1+\beta C^{+}}-\frac{C^{-}}{1+\beta C^{-}}\right)\;.$$

**Figure 5:**
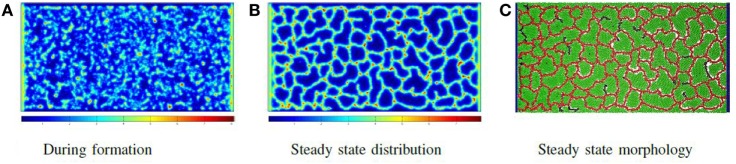
**Phase I: self-organizing the network: the distribution of chemoattractant during vessel formation and at a steady state, along with the final vessel network formed**. In all images of biochemical distribution, blue signifies a low concentration, while red signifies a high concentration **(A–C)**.

The force $F\cdot \vec{c}$ is only applied to the particle if *F* > 0. Once all particles have been assigned forces, the system is relaxed by a shoving algorithm that moves the particles to avoid overlapping. In this way, the vessel particles push through the production particles, form clumps due to attractive adhesive forces and then buckle and extend immature vessels. The system eventually reaches the morphology illustrated in Figure 5C, in which all biomechanical forces are relaxed and concentrations of molecules are stable. Figure 5 shows the chemoattractant distribution during formation and at the stable state, along with the corresponding final cellular morphology.

### 4.2 Phase II: Determine Network Flow

**Figure 6:**
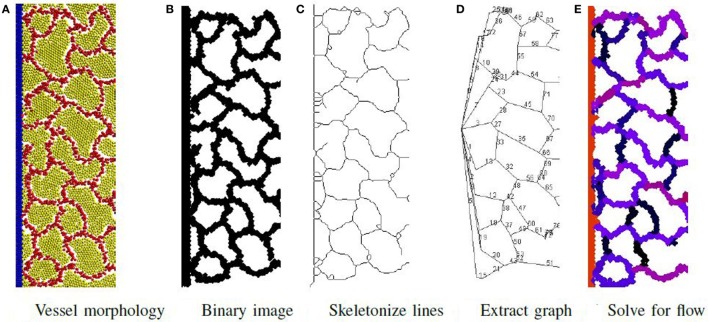
**Phase II: initially, a network of pipes is extracted from the vessel morphology in steps (A–D)**. Then, the flow rate of material through each vessel is determined and illustrated **(E)** using a color map to signify flow rate magnitude. Red is highest flow rate and black is the lowest.

The process to determine material flow through the network is illustrated in Figure 6 and consists of first extracting a network-of-pipes representation of the cellular morphology, then simulating its execution. To identify the pipes and their connectivity, the output file produced by the simulation is visualized as an image using POV-Ray software (Persistence of Vision Pty. Ltd, 2004). Particles are rendered as illuminated spheres of differing colors that signify their type as illustrated in Figure 6A. This image is then converted to a binary image shown in Figure 6B, and the local width of each vessel is extracted using Fiji software (Schindelin et al., 2012), an extension of ImageJ (Schneider et al., 2012; Schindelin et al., 2015). The local width measure is needed during the last step of the process. The binary image is skeletonized using the algorithm of Lee et al. (1994) (illustrated in Figure 6C) and implemented by the Ignacio Arganda-Carreras software (Arganda-Carreras et al., 2010). The skeletonized image is transformed to a skeletonized graph (see Figure 6D) using the AnalyzeSkeleton algorithm (Arganda-Carreras et al., 2010). This skeletonized graph is planar with each edge representing a unique vessel in the network. Each edge is assigned a radius (half the average local width) and a length. The network is now represented as a graph of pipes with *n* nodes and *m* vessels and is ready for the final step.

First, a graph traversal is performed to determine if a path exists through the network connecting the source (the upper left) and sink (lower right) in Figure 1B. Each node *i* in the graph is assigned an unknown variable *P_i_* representing the pressure at node *i*. Each edge (*i, j*) in the graph is assigned an unknown variable *Q*_(*i,j*)_ representing the flow through *i, j*, and an unknown variable Δ*P*_(*i,j*)_, representing the pressure drop *P_i_* − *P_j_*. Next a series of equations of the graph are generated using Poiseuille’s Law (Sutera and Skalak, 1993) as given in equations (3)–(5), relating *Q*_(*i,j*)_, Δ*P*_(*i,j*)_, and *P_i_* to characteristics of the network (*r* and *l* the radius and length of each vessel) and operating conditions (*P*_1_ and *P_n_*, the source and sink pressure, respectively). For this simulation, Δ*P*_(1,*n*)_ was set at 1 KPa (Wilking et al., 2013). The viscosity of the fluid η is set to that of water at 25°C. The following linear equations are generated:

For each edge *i, j*,

(3) $$Q_{\left(i,j\right)}=\frac{\pi r^{4}\Delta P_{\left(i,j\right)}}{8\eta l}.$$

For each node, *i* with σ(*i*) neighbors,

(4) $$\sum_{k\in \sigma \left(i\right)}\;Q_{\left(i,k\right)}=0.$$

For each lacunae cycle ϕ(*k*),

(5) $$\sum_{\left(i,j\right)\in \phi \left(k\right)}\;\Delta P_{\left(i,j\right)}=0.$$

Finally, these equations are solved using a linear equation solver to calculate the flow rate *Q*_(*i,j*)_ through all the vessels and the pressure drop Δ*P*_(*i,j*)_ over each vessel. The final solution is illustrated in Figure 6E with a color map signifying the magnitude of the flow. Note that the vessels in the horizontal direction tend to have high flow, while the vessels in the vertical direction tend to have low flow.

### 4.3 Phase III: Run the Factory

To execute the factory, each vessel provides nutrient *N* and removes product *X* along the vessel length. The rate of *N* and *X* through a vessel increases with flow rate and radius but is limited by transfer rates through the vessel walls (Chrispeels et al., 1999). Models have been developed for engineered vascular networks in the study by Morin et al. (2015) and *in vitro* networks in the study by O’Dea et al. (2015). This work employs a simplified model described below.

The rate of nutrient supply along the vessel (*i, j*) is defined as:

(6) $$\frac{\partial N_{\left(i,j\right)}}{\partial t}=\rho_{n}2\pi r\frac{Q_{\left(i,j\right)}}{\left(k_{\mathrm{out}}+Q_{\left(i,j\right)}\right)}\frac{k_{l}}{\left(k_{l}+N\right)},$$

where *Q*_(*i,j*)_ is the flow rate determined from Phase II, ρ*_n_* is a transfer constant, and *r* is the radius of the vessel. The rate of nutrient delivery is controlled by the flow in the vessel, represented as a Hill function of *Q*_(*i,j*)_, and the amount of *N* in the microenvironment. As the nutrient in microenvironment decreases, the rate of nutrient delivery increases, defined in the function above.

The availability of nutrient will activate the producer cells that will begin to consume *N* and produce *X*. The controlling equation is given as

(7) $$\frac{\partial X}{\partial t}=\mu_{p}\frac{N}{\left(N+k_{p}\right)}\frac{k_{i}}{\left(X+k_{i}\right)}M_{p}+D_{p}{▿}^{2}X,$$

where *M_p_* is producer cell biomass. To produce a realistic model of production, the parameter values of the producer cells is replicated from the study by Bernard et al. (1999) and is based on vanillin production of *Pycnoporus cinnabarinus*. The vascular cell factory approach is not specific to this cell type and product. Other models of producer cells may be substituted. The product *X* is secreted by producer cells consuming *N* following Michaelis–Menten kinetics with a reaction rate of μ*_p_*, where the saturation of enzymes involved in the *X* production is considered *k_p_*. The negative feedback due to product inhibition is also taken into account, with a correspondent inhibitor constant *k_i_* (Aiba et al., 1968; Levenspiel, 1980; Han and Levenspiel, 1988).

The rate of product uptake along the vessel (*i, j*) is defined as:

(8) $$\frac{{\partial X}_{\left(i,j\right)}}{\partial t}=-\rho_{p}2\pi r\frac{Q_{\left(i,j\right)}}{\left(k_{\mathrm{in}}+Q_{\left(i,j\right)}\right)}\frac{X}{\left(k_{p}+X\right)}$$

where *Q*_(*i,j*)_ is the flow rate determined from Phase II, ρ*_p_* is a transfer constant, and *r* is the vessel radius. The rate is controlled by the flow similarly to *N*, but in this case, the effect of *X* in the microenvironment is different. Here, as the amount of *X* increases, the rate of removal increases.

Nutrient will be consumed by the producer cells in direct correspondence to the production of *X*, but at a different reaction rate μ*_n_*:

(9) $$\frac{\partial N}{\partial t}=-\mu_{n}\frac{N}{\left(N+k_{p}\right)}\frac{k_{i}}{\left(k_{i}+X\right)}M_{p}+D_{p}{▿}^{2}N.$$

**Figure 7:**
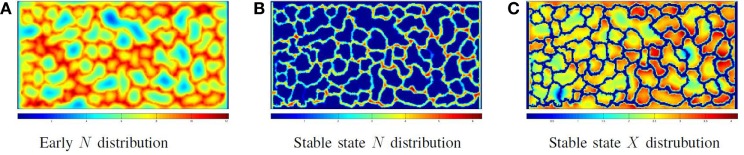
**Phase III: running the factory: the distribution of nutrient (A) when the vessel flow begins, but the producer cells are not yet active and (B) at steady-state nutrient distribution when the factory is running; note that all extravessel nutrient is consumed**. **(C)** The product distribution when the factory is running.

Figure 7 illustrates the cellular factory producing product from nutrient flow for the network illustrated in Figure 5C. Figure 7A illustrates a solution to equations (6) and (9) before producer cells are active. Figure 7B illustrates the nutrient distribution following producer cell activation. In Figure 7C, the distribution of product is illustrated. Note the regions of low product (the blue areas) where vascular flow is limited.

The final step is to extract the product from the fluid flowing out of the factory. This fluid will contain product, unused nutrient, and waste. The method utilized for separation is product dependent, but methods developed for conventional bioreactors will be equally applicable to vascular factories.

## 5 Discussion and Conclusion

This work presents a computational demonstration of a possible vascular factory design. The potential increases in cell factory productivity by a self-organizing vascular system were assessed through an integrated model of vessel morphogenesis and dynamic vascular system functioning. The robustness of vessel self-organization was evaluated by a stochastic model over a population of random initial states, and scalability was assessed by varying the separation distance between circulatory vessels.

Bioreactor design has remained stagnant for the past decade. The greatest improvement has come from the introduction of single-use bioreactors, which feature disposable large bags containing presterilized and mixed media that are agitated (Shukla and Gottschalk, 2013). However, the general bioreactor design remains the same. In the investigations described here, the use of simulated vascular factories fundamentally shifts how bioreactors can be used to grow healthy cells and generate valuable products. Vascularized factories do not rely on stirring or shaking. Instead, the formation of well-defined vascular networks feeds the cells, eliminates waste, and protects the products. Furthermore, there is no explicit engineering design needed to construct the vascular network because it is formed by self-organizing endothelial-like cells.

To move these cellular factories from a computational model to a real-world tool, specific cell types must be engineered that exhibit the requisite properties, such as chemotaxis, and be able to function together to create a stable tissue. Recent work in engineering-induced pluripotent stem cells may hold the key to advances in this area (Warren et al., 2010; Robinton and Daley, 2012), particularly for vascular systems (Leeper et al., 2010). In addition, a means must be devised for linking the nascent vessels with the preexisting circulatory system. Here, advances in microfabrication may be relevant (Borenstein et al., 2002).

For insights provided by simulations to be useful, simulated cells must accurately reflect cell physiology and the underlying biomechanical and biochemical physiological processes. Recent multiscale models have advanced tissue modeling and cell model validation techniques in cancer and (Macklin et al., 2012) age-related macular degeneration (Baker et al., 2015). Such techniques can be applied to improve the fidelity of vascular factory models.

Optimization of vascular bioreactors will require millions of simulations and necessitate a significant speedup of simulator execution time. In addition to rapid execution, the scale of the simulations needs to be expanded to billions of cells to consider cell factories in three dimensions. Such 3D designs hold promise for significant improvements because the vessels could support far more producer cells. Recently, two fast large-scale simulation systems have been developed by Ghaffarizadeh et al. (2015) and *Biocellion* (Kang et al., 2014). Both these systems implement an individual-based approach similar to *cDynoMiCs* employed here. *Biocellion* is implemented as a distributed architecture executable on the Cloud (Ibrahim et al., 2015) and is capable of simulating complex 3D models of billions of cells in a matter of a few hours. *Biocellion* has the potential to simulate an industrial-scale vascular microbial cell factory consisting of trillions of cells.

In summary, this simulation study has demonstrated that vascular cell factories have the potential to be robust and scalable, leading to significant increases in productivity and changes to bioreactor designs. Advances in cellular and tissue engineering will be needed to implement such a design, and major progress is constantly occurring. Implementation of this type of cell factories may fundamentally change the way pharmaceutical and high-value biological items are produced.

## Author Contributions

NF conceived the idea and directed the design, implementation, evaluation, and writing. DD implemented the model in software, performed all the experiments, and contributed to the writing. AD helped develop the biological model and assisted in the writing. GP consulted on all biological aspects of the model and led the writing effort. EV assisted in model development and contributed to the writing.

## Conflict of Interest Statement

The authors declare that the research was conducted in the absence of any commercial or financial relationships that could be construed as a potential conflict of interest.
